# Porcine Coronin 1A Contributes to Nuclear Factor-Kappa B (NF-κB) Inactivation during *Haemophilus parasuis* Infection

**DOI:** 10.1371/journal.pone.0103904

**Published:** 2014-08-05

**Authors:** Chong Liu, Yang Wang, Hengling Zhang, Shuang Cheng, Catherine Charreyre, Jean Christophe Audonnet, Pin Chen, Qigai He

**Affiliations:** 1 Division of Animal Infectious Diseases, State key Laboratory of Agricultural Microbiology, College of Veterinary Medicine, Huazhong Agricultural University, Wuhan, P. R. China; 2 State key Laboratory of Veterinary Biotechnology, Harbin Veterinary Research Institute, Chinese Academy of Agricultural Sciences, Harbin, P. R. China; 3 Merial SAS, Lyon, France; Cornell University, United States of America

## Abstract

*Haemophilus parasuis* (*H.parasuis*) is the etiological agent of porcine polyserositis and arthritis (Glässer's disease) characterized by fibrinous polyserositis, meningitis and polyarthritis, causing severe economic losses to the swine industry. Currently, the molecular basis of this infection is largely unkonwn. Coronin 1A (Coro1A) plays important roles in host against bacterial infection, yet little is known about porcine Coro1A. In this study, we investigated the molecular characterization of porcine Coro1A, revealing that porcine Coro1A was widely expressed in different tissues. Coro1A could be induced by lipopolysaccharide (LPS), polyinosinic acid-polycytidylic acid [poly (I:C)] and *H.parasuis* in porcine kidney-15 (PK-15) cells. Functional analyses revealed that porcine Coro1A suppressed the NF-κB activation during *H.parasuis* infection by inhibiting the degradation of IκBα and nuclear translocation of p65. Overexpression of porcine Coro1A inhibited the transcription of NF-κB-mediated downstream genes [Interleukin-6 (IL-6), Interleukin-8 (IL-8) and COX-2] through down-regulation of NF-κB. The results indicated that porcine Coro1A is an important immunity related gene that helps to inhibit NF-kB activation during *H. parasuis* infection.

## Introduction

Coronin was firstly isolated from Dictyostelium discoideum and localized to crown-like structures on the apical surface of the cells [1]. To date, 7 members of the mammalian coronin family have been found, as follows: Coronin 1–5 [2], Coronin 6 (cor_SE_) and Coronin 7/POD [3]. Coronins belong to the WD (Trp-Asp) repeat superfamily, which are found in all eukaryotes and have been highly conserved during evolution [3]–[5]. A defining structural characteristic of coronins is the presence of an N-terminal WD40 repeat domain, separated by a unique region [6]. Moreover, WD-repeats are involved in vital biological processes, such as cytoskeletal organization, signal transduction and membrane trafficking [7]. Coronins mainly associate with the membrane cytoskeleton through interaction with F-actin and the Arp2/3 complex, regulating cell motility and cytoskeletal rearrangement [8], [9].

Coro1A, a member of mammalian coronin family, also known as Tryptophan Aspartate containing coat protein (TACO) or p57 [10], is mainly expressed in hematopoietic cells and equally distributes between the cytosol and the cell cortex [11]. It has been demonstrated that Coro1A plays a crucial role in T lymphocyte activation [11], [12], migration [13], survival [14], and calcium signal transduction [15]. In neutrophils, Coro1A associates with actin and the soluble component p40^phox^ subunit of the NADPH oxidase complex [16], [17]. While in macrophages, Coro1A associates with phagosomes [18], [19]. Previous study has clarified the association between human Coro1A and the intracellular survival of pathogenic mycobacterium [20], [21].

The Gram-negative bacterium *Haemophilus parasuis* (*H.parasuis*) has generated a worldwide threat to the swine herds, and leads to a huge economic loss to the pig industry. 15 serovars of *H.parasuis* have been identified so far [22]. The virulent strains of *H.parasuis* are the causative agent of Glässer's disease, and the disease is characterized by fibrinous polyserositis, meningitis and polyarthritis [23], all of which are associated with uncontrolled inflammation. Besides, acute pneumonia, acute septicemia, acute fasciitis and myositis happened occasionally [24]. The pathogenesis of *H.parasuis* infection is still unknown. *Chen et al* (2012) reported that *H.parasuis* could activate the NF-κB pathway in PK-15 cells [25]. The NF-κB signaling pathway is important in signal transduction during the innate immune response [26]. NF-κB signaling relies on the targeting of IκB (inhibitor of NF-κB) subunit to the proteasome to allow NF-κB to translocate from the cytosol to the nucleus where it activates transcription of pro-inflammatory cytokine genes, which are essential to mount a protective immune response and host defence [27]. In our previous study, we found porcine Coro1A was differentially expressed in *H.parasuis* infected porcine alveolar macrophages, which were considered as a major component of the host innate immunity [28]. The innate immune response in vertebrates is the first defense line against invading microorganisms. Interestingly, human Coro1A is mentioned as a novel inhibitor of TLR-mediated NF-κB activation in *Mycobacterium leprae* infection [18]. So in this study, we explored the immunological functions of porcine Coro1A during *H.parasuis* infection. The results indicated that porcine Coro1A is an important immunity related gene that helps to inhibit NF-κB activation during *H.parasuis* infection.

## Materials and Methods

### Ethics statement

All of the animal experiments were approved by the Research Ethics Committee of College of Veterinary Medicine, Huazhong Agricultural University, Hubei, China (No. 2009–0012).

### Cells, bacteria and reagents

PK-15 cells (ATCC number CCL-33; American Type Culture Collection) were cultured and maintained in Dulbecco's Modified Eagle's Medium (DMEM) supplemented with 10% heat-inactivated newborn calf serum (NCS), 100 U/ml penicillin, and 10 µg/ml streptomycin sulfate.

*H.parasuis* 0165 strain was clinically isolated in China with high-level virulence. Bacteria were cultured in tryptic soy broth (TSB; Difco) supplemented with 10 mg/ml nicotinamide adenine dinucleotide (NAD; Sigma) and 5% fetal bovine serum (Gibco), Bacteria were harvested by centrifugation at 5000 g for 5 min, washed three times with sterile PBS and resuspended in PBS. Heat-killed bacteria were prepared by heat treatment for 20 min at 70°C.

TNF-α, LPS and poly (I:C) were purchased from Sigma-Aldrich (St.Louis, MO, USA). Monoclonal antibodies anti-IκBα, p65, phospho-p65, β-actin and polyclonal antibody anti-Histone H3 (1∶1000 diluted) were purchased from Cell Signaling Technology (USA). Horseradish peroxidase-conjugated anti-mouse or anti-rabbit IgG (1∶1000 diluted) were purchased from Beyotime Institute of Biotechnology (Jiangsu, China). The luciferase reporter plasmid pNF-κB-Luc contains four repeats of κB binding motifs followed by the luciferase reporter gene (Luc) and the internal control plasmid pRL-TK were purchased from Stratagene (USA).

### Porcine Coronin 1A gene expression and protein purification

The porcine Coro1A gene was amplified from cDNA of *H.parasuis* infected Porcine alveolar macrophages (PAM) by PCR primed with the following primer pair: the forward primer 5′-TTT GAA TTC ATG AGC CGG CAG GTG GTC C-3′ and the reverse primer 5′-GGG GAA GCT TCT ACT TGG CCT GGA CTG TC-3′. The PCR product was cloned into the TA cloning vector and pET-30a plasmid, respectively (TakaRa Bio Inc., Otsu, Shiga, Japan). For sequencing the DNA of porcine Coro1A, the PCR product was inserted into the TA cloning vector (TakaRa), according to the instructions of the manufacturer. The ligation product was used to transform into *E.coli* DH5α Positive clones contaning the porcine Coro1A gene were sequenced. For the protein expression, the PCR product was inserted into the pET-30a vector, recombinant plasmids were transformed into *E.coli* BL_21_ (DE3) cells to obtain the recombinant fusion protein designated His-rPoCoro1A. His-tagged recombinant protein purified under non-denaturin conditions (using Ni-NTA His Bind Resin).

### The Porcine Coro1A antibody

Polyclonal antibodies against porcine Coro1A were prepared in female BALB/C mouse by infection with rPoCoro1A protein. The mouse were bled ten days after the third immunization and the antibody titers were measured by enzyme-linked immunesorbent assay (ELISA). Antibodies were purified using Protein A High-Capacity Agarose and Kits (Thermo) and quantified using a BCA Protein Assay Kit (Beyotime Institute of Biothechnology, Nan Tong, China).

### qPCR analysis of porcine Coro1A expression in tissues

Three pigs in *H.parasuis* infection group and control group were selected for the analysis of porcine Coro1A expression in different tissues. Total RNA from 5 porcine organs (inguinal lymph node, heart, spleen, lung, brain) was isolated. For the tissue-specific expression of porcine Coro1A, total cellular RNA was extracted from 15 different swine tissues: heart, liver, spleen, lung, kidney, pancreas, stomach, cerebellum, bowel lymph node, inguinal lymph node, mandibular lymph node, cerebrum, duodenum, tonsil and colon. Total RNA was isolated by using the Simply P Total RNA Extraction Kit (Bioflux), and was reverse-transcribed into cDNA by using ReverTraAce Kit (TOYOBO), according to the manufacturer's protocols. The quantitative real-time PCR (Q-PCR) assay was performed by using LightCycler 480 SYBR Green I Real Time PCR Master Mix (Roche) with primer pairs described in table 1, with the GAPDH was used as the reference gene. The specificity of the primers was confirmed by melting curve analysis.

**Table 1:** Primers used in quantitative real-time PCR.

Gene	Primer sequence
Coro1A	F: 5′-GTGGACTGGAGCCGAGA-3′
	R: 5′-GCCACCTGCCGCTCACTC-3
IL-6	F: 5′-CCTTCAGTCCAGTCGCCTTCTCC-3′
	R: 5′-GCATCACCTTTGGCATCTTCTTCC-3′
IL-8	F:5′-CACTGTGAAAATTCAGAAATCATTGTTA-3
	R: 5′-CTTCACAAATACCTGCACAACCTTC-3′
COX-2	F: 5′-TTCAACCAGCAATTCCAATACCA-3′
	R: 5′-GAAGGCGTCAGGCAGAAG-3′
GAPDH	F: 5′-ACATGGCCTCCAAGGAGTAAGA-3′
	R: 5′-GATCGAGTTGGGGCTGTGACT-3′

### LPS, poly (I:C) and *H.parasuis* induced expression of porcine Coro1A in PK-15 cells

PK-15 cells were seeded at a concentration of 4×10^5^ cells/well into six-well culture plates (Corning) and grown in culture medium (DMEM) supplemented with 10% heat-inactivated newborn calf serum (NCS) at 37°C with 5% CO_2_. Adherent PK-15 cells were washed twice with sterile PBS and cultured further in DMEM and treated with 1 µg/ml LPS, 10 µg/ml poly (I:C) or 10^7^ CFU of *H.parasuis* for 0, 2, 6, 12, 24, and 48 h (three replicates in each group). The cells were harvested at each time, and total RNA was extracted by using Simply P Total RNA Extraction Kit, and RT-PCR was employed by using ReverTraAce Kit. The Q-PCR assays were performed by using LightCycler 480 SYBR Green I Real Time PCR Master Mix with primer pairs described in table 1, and samples were normalized with the samples collected at 0 h as the calibrator and the GAPDH as the reference gene. All Q-PCRs were performed in triplicate.

### Plasmid construction

The coding sequence of porcine Coronin 1A was subcloned into the pcDNA-3.1 vector (Invitrogen,USA) using *Bam*HI/*Eco*RI sites, resulting in the expression constructs pcDNA-Coro1A.

### Transfections and luciferase reporter assays

Transient transfection was performed using Lipofectamine 2000 (Invitrogen,USA). PK-15 cells were seeded on 24-well tissue culture plates (Corning) and cultured until the cells reached approximately 70–80% confluence, and were then transfected with the plasmids listed below. For each transfection, 0.2 µg of the reporter plasmid pNF-κB-Luc along with 0.05 µg of pRL-TK for normalization and expression plasmids or empty control plasmid were used. Firefly and Renilla luciferase activities were measured by using the dual-luciferase reporter assay system (Promega) according to the manufacturer's instructions. Data represent relative firefly luciferase activity.

### Western blot analysis

PK-15 cells were seeded at a concentration of 4×10^5^ cells/well into six-well tissue culture plates until the cells reached approximately 70–80% confluence. Transfection was performed with Lipofectamine2000 reagent following the manufacturer's protocols. At 24 h post-transfection, cells were washed twice with PBS, harvested and lysed, then boiled in SDS protein sample buffer (2% SDS, 10% glycerol, 60 mM Tris-HCl [pH 6.8], 0.001% bromophenol blue, and 0.33% b-mercaptoethanol). Cells infected with live *H.parasuis* for 12 h were treated the same. The cell lysates were separated by 10% acrylamide SDS-PAGE, followed by electroblotting onto a nitrocellulose membrane. Western blots were performed with homemade anti-Coronin 1A polyclonal antibody, anti-IκBα, anti-p65, anti-p-p65, anti-β-actin monoclonal antibodies and anti-Histone H3 polyclonal antibody as well as HRP-conjugated goat anti-mouse or goat anti-rabbit IgG, respectively. Signals were visualized by using SuperSignal West Pio Luminol kit (Pierce). Analysis of bands was performed using the public domain ImageJ program (developed at the National Institute of Health and available at http://rsb.info.nih.gov/ij).

### RNA extraction and real-time quantitative analysis of IL-6, IL-8 and COX-2

Total RNA was extracted from the PK-15 cells by using Simply P Total RNA Extraction Kit, and RT-PCR was employed by using ReverTraAce Kit. The Q-PCR assays were performed by using LightCycler 480 SYBR Green I Real Time PCR Master Mix with primer pairs described in table 1. The housekeeping gene GAPDH was used as an internal control. All Q-PCRs were performed in triplicate.

### Statistical analysis

Data were presented as means ± standard deviations (SD). Statistical analysis was performed by using the Statistical Package Social Sciences (SPSS) program, version 17.0. Student's T-test was used to determine statistical significance. A *p*-value less than 0.05 was considered significant and a *p*-value less than 0.01 was considered highly significant.

## Results

### Porcine Coro1A protein purification and antibody production

**Figure 1:**
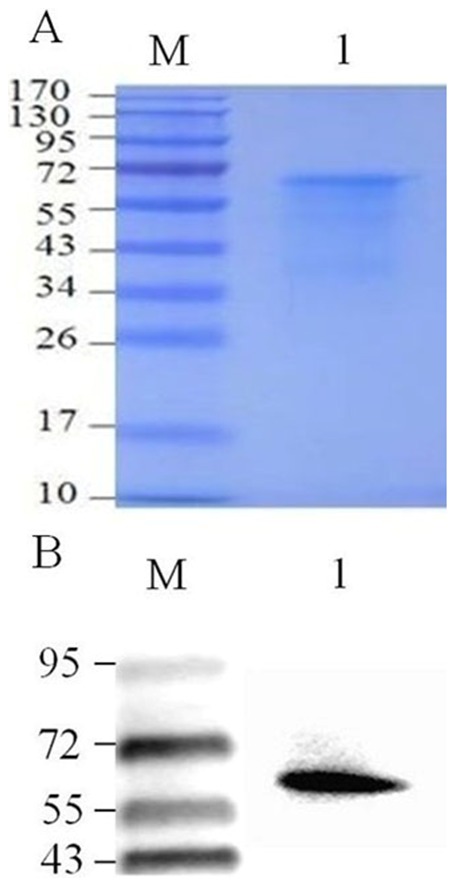
Porcine Coronin1A expression. A. SDS-PAGE analysis of rPoCoro 1A (Lane 1). Lane M, protein molecular weight marker. B. Anti-rPoCoro 1A polyclonal antibodies determination by western blotting. Lane 1, rPoCoro 1A. Lane M, protein molecular weight marker.

The coding sequence of porcine Coro1A comprised 1386 bp with a G+C content of 63.2%. PCR product by amplified porcine Coro1A gene was cloned into pET-30a expression vector and transformed into *E. coli* BL_21_ (DE3) cells. With prokaryotic expression system, we obtained a soluble recombinant protein migrating on SDS-PAGE with a molecular mass about 65 kDa (**Fig. 1A**). This recombinant protein was used to raise specific mouse polyclonal antiserum against porcine Coro1A. The serum specifically reacted with the 65 kDa band of rPoCoro1A (**Fig. 1B**).

### Tissue-specific expression of porcine Coro1A

In order to elucidate the basal expression of porcine Coro1A, Q-PCR [29] was used to analyze its transcript in mRNA prepared from 15 different tissues. Using transcript-specific and consensus region-specific primer pairs described in table 1 and the housekeeping gene GAPDH was used as an internal control.

**Figure 2:**
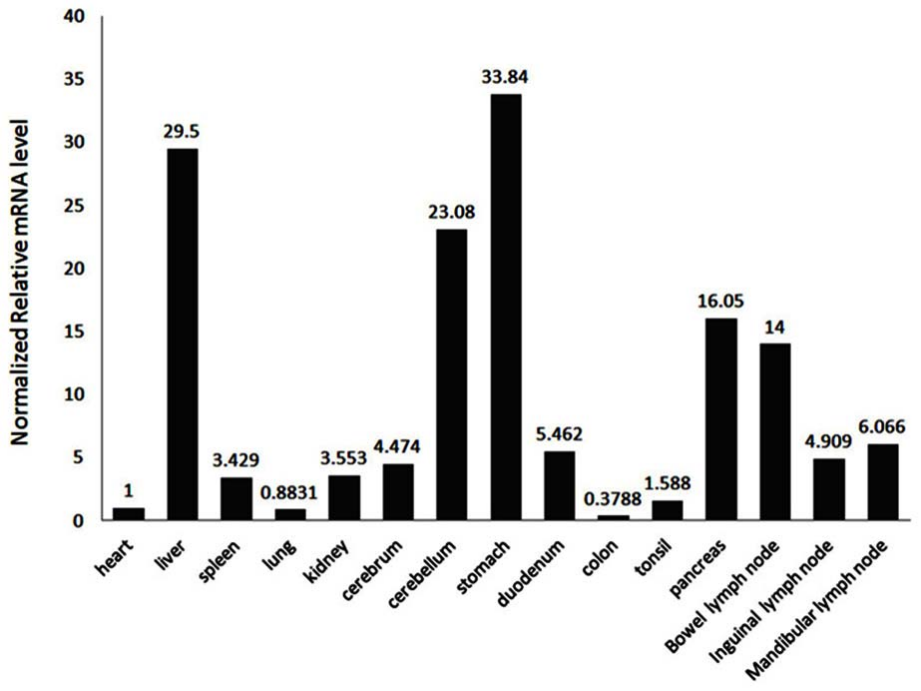
Basal expression of porcine Coro1A in different tissues. The expression of Coro1A was firstly normalized to the expression of GAPDH and then compared relative to the expression of Coro1A in heart, which was set as 1.

As shown in Figure 2, porcine Coro1A was highly expressed in the liver, pancreas, stomach, cerebellum, bowel lymph node and moderately expressed in the inguinal lymph node, mandibular lymph node, cerebrum, kidney, spleen, duodenum, tonsil and lowly expressed in heart, lung, and colon. All in all, porcine Coro1A was found ubiquitously expressed in all examined tissues. Interestingly, the expression level of porcine Coro1A in liver was notably higher than any other investigated tissues, which was different from human and mouse [10], [20]. In addition, although there is contradiction among the different species in Coro1A mRNA expression, abundant Coro1A mRNA expression was observed in the immune organs of human, mouse and pig, indicating that Coro1A might play roles in the mammalian immune system.

### Expression analysis of porcine Coro1A in PK-15 cells stimulated with LPS, poly (I:C) and *H.parasuis*

**Figure 3:**
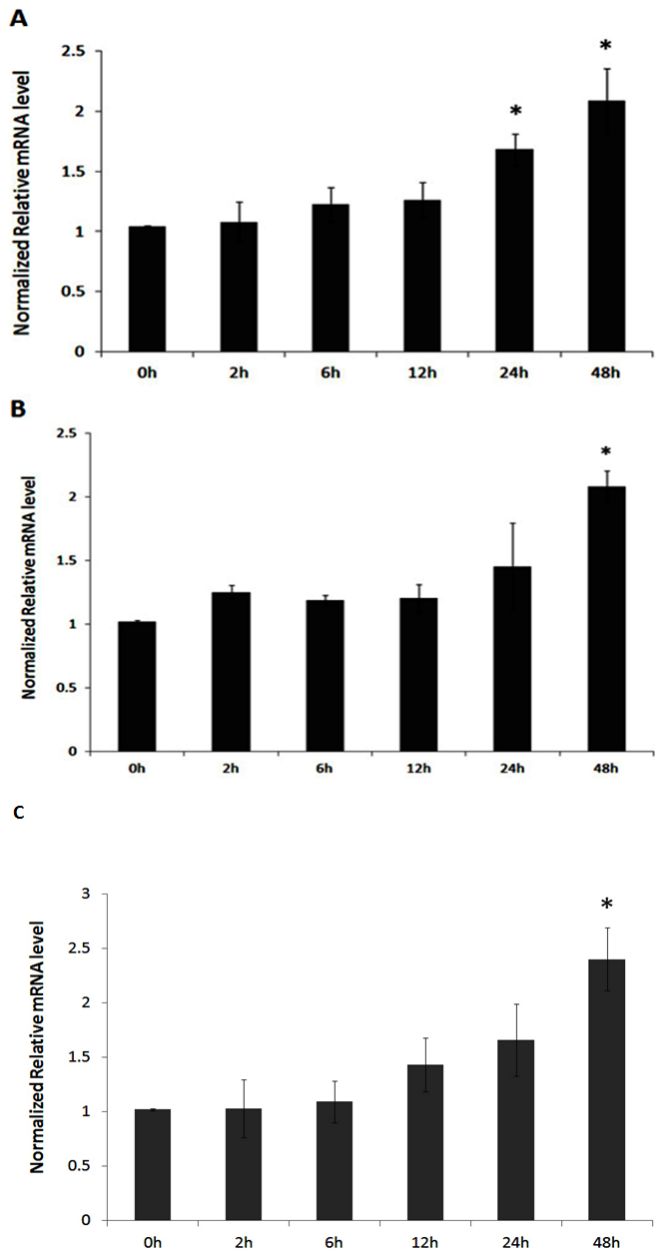
Expression analysis of porcine Coro1A in PK-15 cells stimulated with LPS, poly (I:C) and *H.parasuis*. A: LPS-induced expression of porcine Coro1A in PK-15 cells. PK-15 cells were cultured with 1 µg/ml LPS for 48 h. B: Poly (I:C)-induced expression of porcine Coro1A in PK-15 cells. PK-15 cells were cultured with 10 µg/ml poly (I:C) for 48 h. C: *H.parasuis*-induced expression of porcine Coro1A in PK-15 cells. PK-15 cells were cultured with 10^7^ of CFU *H.parasuis* for 48 h. Relative expression of porcine Coro1A was detected by Q-PCR and normalized to the expression of GAPDH. The fold increase is expressed as the mean of three replicates with SEM by comparison with the control (0 h). Q-PCR was performed using primers described in table 1. Results are from the calculated average ± SD of three different cell samples in the same treatment. *P<0.05.

PK-15 cells have been shown especially useful for the study of infectious disease processes in swine [25], [30]. In order to investigate the expression patterns of porcine Coro1A under general conditions that imitate bacterial and viral infection, the immunostimulation assay was carried out in PK-15 cells using LPS, poly (I:C) and *H.parasuis* as the stimulators. Overnight cultures of PK-15 cells were washed twice with sterile PBS and maintained in DMEM, then treated with 1 µg/ml LPS, 10 µg/ml poly (I:C) or 10^7^ CFU of *H.parasuis* for 0, 2, 6, 12, 24 and 48 h. LPS stimulation induced up-regulation of Coro1A at 24 h, and reached the peak at 48 h (**Fig. 3A**). The up-regulation of Coro1A under poly (I:C) and *H.parasuis* stimulation was also observed (**Fig. 3B and C**). This study indicated that LPS, poly (I:C) and *H.parasuis* can induce the expression of porcine Coro1A in vitro.

### Porcine Coro1A suppresses NF-κB activation in PK-15 cells

**Figure 4:**
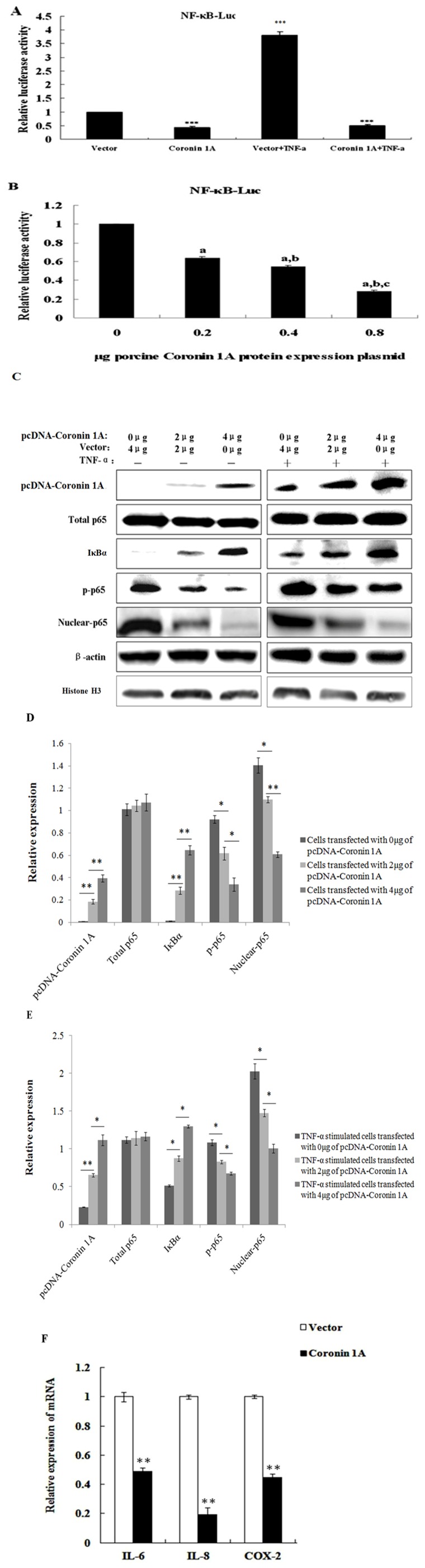
Reduction of NF-κB activation when porcine Coro1A was overexpressed. (A) PK-15 cells were co-transfected with the pNF-κB-luc reporter plasmid (0.2 µg), pRL-TK plasmid (0.05 µg), along with 0.6 µg of the expression plasmid encoding porcine Coro1A protein. Selected cells were stimulated by 20 ng/ml TNF-α at 18 h post-transfection, and cell extracts were prepared for the dual-luciferase activity at 6 h after this treatment. ***P<0.001 as compared with vector control. (B) An increasing quantities of porcine Coro1A expression plasmid (0, 0.2, 0.4, 0.8 µg) was co-transfected with pNF-κB-luc and pRL-TK into PK-15 cells. Cells were harvested at 24 h after transfection and analysed for luciferase activity. (a) *P*<0.05 compared with the vector group, (b) *P*<0.05 compared with 0.2 µg porcine Coro1A protein transfection group, (c) *P*<0.05 compared with 0.4 µg porcine Coro1A protein transfection group. Values for the samples were normalized using Renilla luciferase values and expressed as relative fold change in NF-κB-regulated gene expression compared with vector group, and each untreated empty vector control value was set as a basis level of 1. Data represent means of three replicates and results are representative of at least three independent experiments. (C) Overexpression of porcine Coro1A inhibits the degradation of IκBα and nuclear translocation of p65. PK-15 cells were transfected with the indicated amount (0, 2, 4 µg) of porcine Coro1A expression plasmid. Cell total protein extracts, cytoplasmic extracts and nuclear extracts were prepared at 24 h post-transfection and subjected to western blot analysis with antibodies specific for endogenous IκBα, p65 or p-p65. A polyclonal anti-Coronin 1A antibody was used to confirm the expression of Coro1A. The β-actin (for porcine Coro 1A, IκBα, total p65, p-p65 samples) and Histone H3 (for nuclear-p65 samples) were respectively used as a control for sample loading. Similarly, cells were treated with TNF-α (20 ng/ml) at 18 h post-transfection, and cell total protein extracts, cytoplasmic extracts and nuclear extracts were prepared for the western blot analysis at 6 h after this treatment. Western blot analyses were repeated in three independent experiments with similar results and a representative blot was selected. (D) Band densitometry was performed on the western blot shown in figure 4C left. The β-actin (for porcine Coro 1A, IκBα, total p65, p-p65 samples) and Histone H3 (for nuclear-p65 samples) were respectively used for normalization (*P<0.05, **P<0.01, Student's T-test). (E) Band densitometry was performed on the western blot shown in figure 4C right. The β-actin (for porcine Coro 1A, IκBα, total p65, p-p65 samples) and Histone H3 (for nuclear-p65 samples) were respectively used for normalization (*P<0.05, **P<0.01, Student's T-test). (F) PK-15 cells were transfected with 4 µg of plasmid encoding porcine Coro1A as well as empty vector pcDNA-3.1. At 24 h post-transfection, total RNA was extracted and the relative expression of IL-6, IL-8, and COX-2 genes were evaluated by Q-PCR. Results are expressed as decreasing mRNA levels relative to those in cells transfected with the empty vector and were normalized to the expression of housekeeping gene GAPDH. All data represent the means and standard deviation of three independent experiments. **P<0.01 as compared with empty vector control.

In order to investigate the molecular function of porcine Coro1A in innate immunity, the expression plasmid pcDNA-Coro1A was used for a luciferase reporter assay. As shown in Figure 4A, overexpression of Coro1A in PK-15 cells potently suppressed NF-κB activation. To further confirm the effect of Coro1A on NF-κB, PK-15 cells were transfected with the increasing amounts of the expression plasmid pcDNA-Coro1A, luciferase activity was monitored at 24 h post-transfection. As shown in Figure 4B, a dose-dependent decrease in luciferase reporter activity was observed. These data clarified that porcine Coro1A was responsible for the inhibition of NF-κB activation in PK-15 cells.

### Overexpression of porcine Coro1A inhibits the degradation of IκBα and nuclear translocation of p65

In order to investigate the potential mechanism of NF-κB inactivation by porcine Coro1A, we firstly examined the IκBα expression level in the cytoplasmic extracts using a western blot assay. As shown in Figure 4C and D, the IκBα protein significantly increased in a dose-dependent manner in PK-15 cells transfected with pcDNA-Coro1A. To further characterize the mechanism, the phosphorylation and nuclear translocation of the p65 subunit, as well as the total p65 in Coro1A expression cells were determined. The results revealed that the amount of phosphorylated p65 and nuclear p65 protein significantly decreased in a dose-dependent manner, while the amount of total p65 was unaltered (**Fig. 4C and D**). Because TNF-α is a classic cytokine that induces NF-κB activation, it was possible that Coro1A influences the action of TNF-α. Indeed, porcine Coro1A suppressed TNF-α-induced NF-κB activation in a dose-dependent manner (**Fig. 4C and E**).

### Porcine Coro1A induces down-regulation of NF-κB-regulated gene expression

NF-κB is a critical transcription factor regulating the transcription and expression of many pro-inflammatory molecules, including critical enzymes (for example, COX-2), most cytokines (for examples, IL-6), and chemokines (for examples, IL-8). To further investigate the possible biological impact of porcine Coro1A on pro-inflammatory molecules, the Q-PCR was used to determine if porcine Coro1A inhibited NF-κB regulated target gene expression, including IL-6, IL-8, and COX-2. As shown in Figure 4F, overexpression of porcine Coro1A suppressed the relative expression of IL-6, IL-8 and COX-2. Therefore, we deduced that porcine Coro1A could induce down-regulation of NF-κB-regulated gene expression.

### Porcine Coro1A is a key molecule that modulate NF-κB activation during *H.parasuis* infection

**Figure 5:**
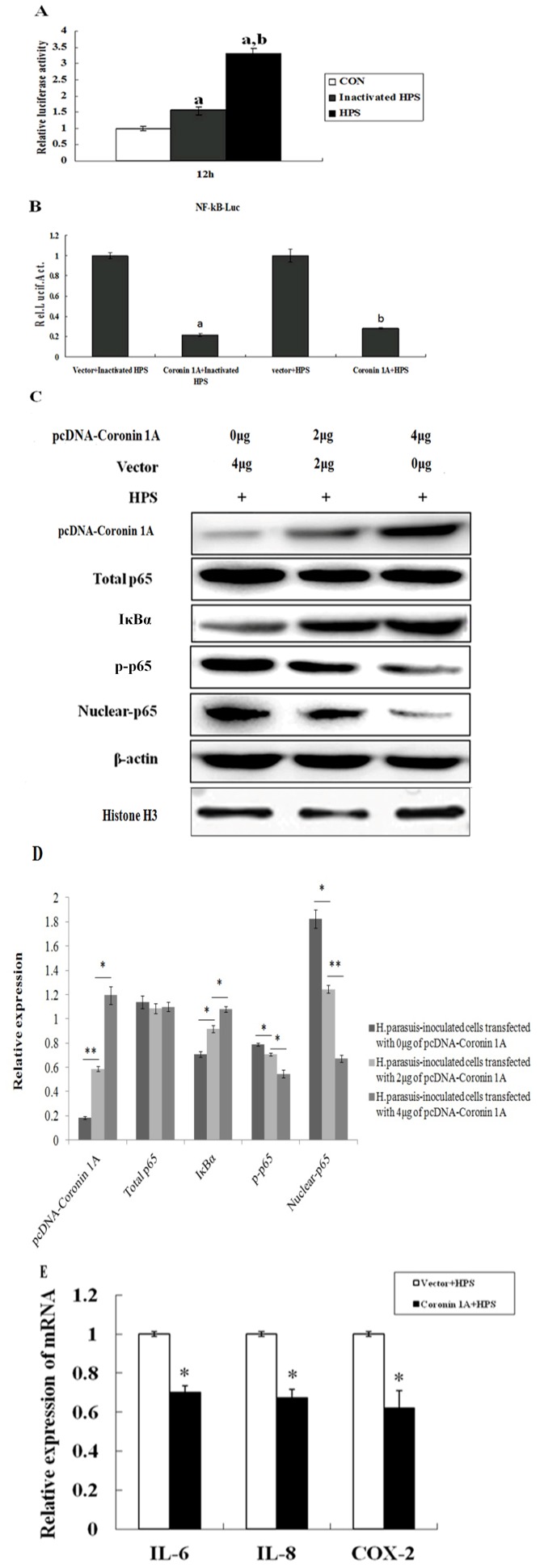
Porcine Coro1A inhibit *H.parasuis* induced NF-κB activation. (A) PK-15 cells were transfected with pNF-κB-luc (0.2 µg) and pRL-TK (0.05 µg), then cells were control-inoculated, live or heat-killed *H.parasuis* (10^7^ CFU) inoculated. (a) *P*<0.05 compared with the control group, (b) *P*<0.05 compared with the inactivated group. (B) PK-15 cells were transfected with 2 µg of plasmid encoding porcine Coro1A as well as empty vector pcDNA-3.1, along with pNF-κB-luc (0.2 µg) and pRL-TK (0.05 µg). At 24 h post-transfection, cells were treated with live or heat-killed *H.parasuis* (10^7^ CFU), and cell extracts were prepared for the luciferase reporter assays at 12 h after this treatment. Values for the samples were normalized using Renilla luciferase values. Inactivated *H.parasuis*-inoculated cells transfected with empty vector value and live *H.parasuis*-inoculated cells transfected with empty vector value were respectively set as a basis level of 1. (a) *P<0.01* as compared with inactivated *H.parasuis*-inoculated cells transfected with empty vector. (b) *P<0.01* as compared with live *H.parasuis*-inoculated cells transfected with empty vector. (C) PK-15 cells were transfected with the varying amount (0, 2, 4 µg) of pcDNA-Coro1A. At 24 h post-transfection, cells were inoculated with live *H.parasuis* (10^7^ CFU) for 12 h. Western blot analysis with antibodies specific for endogenous IκBα, p65 or p-p65 were performed. A polyclonal anti-Coronin 1A antibody was used to confirm the expression of Coro1A. The β-actin (for porcine Coro 1A, IκBα, total p65, p-p65 samples) and Histone H3 (for nuclear-p65 samples) were respectively used as a control for sample loading. Western blot analyses were repeated in three independent experiments with similar results and a representative blot was selected. (D) Band densitometry was performed on the western blot shown in figure 5C. The β-actin (for porcine Coro 1A, IκBα, total p65, p-p65 samples) and Histone H3 (for nuclear-p65 samples) were respectively used for normalization (*P<0.05, **P<0.01, Student's T-test). (E) PK-15 cells were transfected with 4 µg of pcDNA-Coro1A as well as empty vector pcDNA-3.1. At 24 h post-transfection, cells were inoculated with live *H.parasuis* at 10^7^ CFU and then harvested at 12 h for RNA extraction. The levels of IL-6, IL-8 and COX-2 mRNAs were measured by Q-PCR analysis. Results are expressed as decreasing mRNA levels relative to those inoculated *H.parasuis* cells transfected with the empty vector *P<0.05 as compared with *H.parasuis*-inoculated cells transfected with empty vector.

A recent study demonstrated that *H.parasuis* infection in PK-15 cells could induce the NF-κB activation via IκB degradation and the phosphorylation of p65, which allows NF-κB to stimulate expression of target genes associated with variuos inflammations [25]. In order to investigate whether porcine Coro1A is a key molecule that modulate NF-κB activation during *H.parasuis* infection, at 24 h post-transfection of pcDNA-Coro 1A, cells were infected with live or heat-killed *H.parasuis*. Luciferase activities in both live and heat-killed *H.parasuis* infections were monitored at 12 h post-infection, followed by the examination of the IκBα, p65, nuclear p65 and p-p65 expression level using western blot assay. Q-PCR was used to determine the NF-κB regulated gene expression, including IL-6, IL-8, and COX-2. As shown in Figure 5A, both live and heat-killed *H.parasuis* infections could activate the NF-κB in PK-15 cells. Interestingly, overexpression of Coro1A in PK-15 cells consistently suppressed NF-κB activation by inhibiting IκB degradation and nuclear translocation of p65, in spite of *H.parasuis* infections (**Fig. 5B–D**). Furthermore, the relative expression of IL-6, IL-8, and COX-2 was also suppressed (**Fig. 5E**). These data certified that porcine Coro1A could modulate the inflammatory response by regulating the NF-κB signaling pathway, which triggered by *H.parasuis* infection.

### In vivo expression of porcine Coro 1A in pigs with systemic infection of *H.parasuis*

**Figure 6:**
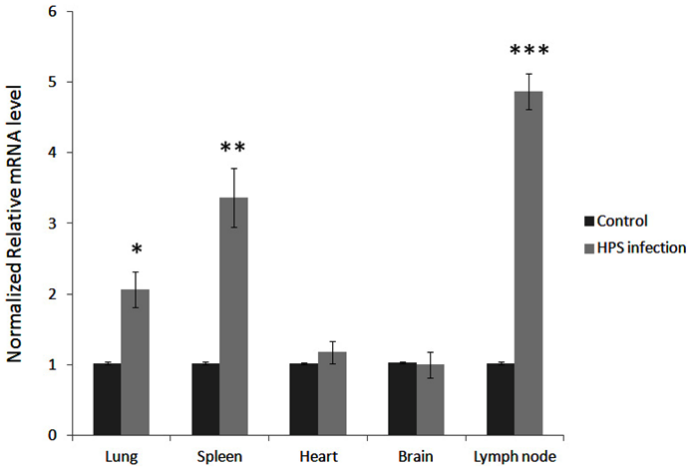
Quantitative expression of porcine Coro1A in five tissues from pigs with Glässer's disease. Increased in vivo gene expression of porcine Coro1A in lungs, spleen, lymph nodes of pigs with Glässer's disease. Relative expression of porcine Coro1A was detected by qPCR and normalized to the expression of GAPDH. The fold increase is expressed as the mean of three replicates with SEM by comparison with the control. The significance of difference for the expression compared to the control was calculated using Student's T-test. ***p<*0.001*; **p<0.01; *p<0.05.

In order to understand the expression of the porcine Coro 1A in pigs with systemic infection of *H.parasuis*, the different tissues obtained from the *H.parasuis* infected pigs and the controls were selected for the qPCR analysis. Our qPCR examination demonstrated that the increasing expression of porcine Coro 1A was observed in the lungs, spleen and lymph nodes of pigs infected with *H.parasuis* (**Fig. 6**). However, in brain and heart of *H.parasuis* infected pigs, the expression of porcine Coro 1A did not show significant changes compared to the controls.

## Discussion

A recent study has indicated that the differentially expressed of Coro1A was observed both in bacterial and viral infections [28], [31]–[33]. However, the molecular characterization of porcine Coro1A and its potential roles in porcine infectious diseases have not been studied.

PK-15 cells have been shown especially useful for the study of infectious disease processes in swine including *H.parasuis* infection [25]. In this study, Coro1A could be induced by LPS, Poly(I:C) and *H.parasuis* in PK-15 cells in vitro. Interestingly, some *H.parasuis* infection related genes were also induced by LPS and Poly (I:C) in PK-15 cells [29], [34]. As classical pathogen-associated molecular patterns, LPS and poly (I:C) were widely used to simulate bacterial and viral infection, respectively, for animal immune response induction [35]. A previous study identified that Coro1A suppressed both LPS-induced NF-κB activation through TLR-4 and poly (I:C)-induced NF-κB activation through TLR-3 [18]. Our data clarified that both LPS and poly (I:C) can induce the expression of porcine Coro1A in vitro, suggesting that porcine Coronin 1A may play roles in host immune system against bacterial and viral infection.

Glässer's disease caused by *H.parasuis* is characterized by a severe inflammation of the serous membranes, including pleuritis, pericarditis and peritonitis, along with meningitis, arthritis and pneumonia [24]. *H.parasuis* diffusely distributed within the mononuclear cells in brains exudate of pigs with fibrinopurulent meningitis suggested the inflammation of serous membranes was associated with inflammatory cells by secreting various cytokines and chemokines [36]. Furthermore, it was reported that *H.parasuis* or its lipooligosaccharide could stimulate IL-8 and IL-6 released by newborn pig tracheal cells and porcine brain microvascular endothelia cells [37], [38]. All these chemokines and cytokines reported involved in initiating adaptive immune responses by NF-κB, which has been shown to play a critical role in regulating the expression of large numbers of genes involved in inflammatory responses [39], [40]. *Chen et al* (2012) reported that *H.parasuis* infection activate the NF-κB via IκB degradation and the phosphorylation of p65 in PK-15 cells, which allowed NF-κB to stimualte expression of target genes associated with various inflammations [25]. During *H.parasuis* infection, the powerful inflammatory response could help host to clear this pathogen or recruite additional inflammatory cells to the site of infection. However, sustained or excessive production of inflammatory cytokines can have damaging consequences. To counterbalance inflammatory cytokines, anti-inflammatory cytokines are produced. *Wilkinson et al* (2010) reported that the IL-1β and its antagonist, IL-1RA are both more highly expressed in “susceptible” animals challenged with *H.parasuis* [41]. A recent study showed that the anti-inflammatory cytokine TGF-β was increased in *H.parasuis* infected PAMs [28]. Anti-inflammatory cytokines may decrease the potentially damaging effects of proinflammatory cytokine on host tissue. Another molecules that performed anti-inflammatory effects were by modulating NF-κB activation [42]. In this study, we proved porcine Coro1A was a novel innate immunity related gene that contributes to inhibit NF-κB activation during *H.parasuis* infection by inhibiting the degradation of IκBα and nuclear translocation of p65. This study may help us to understand the complex mechanisms of host immunity response during *H.parasuis* infection.

## References

[1] de Hostos EL, Bradtke B, Lottspeich F, Guggenheim R, Gerisch G (1991) Coronin, an actin binding protein of Dictyostelium discoideum localized to cell surface projections, has sequence similarities to G protein beta subunits. EMBO J 10: 4097–4104. 1661669 10.1002/j.1460-2075.1991.tb04986.x PMC453159

[2] de Hostos EL (1999) The coronin family of actin-associated proteins. Trends Cell Biol 9: 345–350. 10461187 10.1016/s0962-8924(99)01620-7

[3] Uetrecht AC, Bear JE (2006) Coronins: the return of the crown. Trends Cell Biol 16: 421–426. 16806932 10.1016/j.tcb.2006.06.002

[4] Xavier CP, Eichinger L, Fernandez MP, Morgan RO, Clemen CS (2008) Evolutionary and functional diversity of coronin proteins. Subcell Biochem 48: 98–109. 18925374 10.1007/978-0-387-09595-0_9

[5] Roadcap DW, Clemen CS, Bear JE (2008) The role of mammalian coronins in development and disease. Subcell Biochem 48: 124–135. 18925377 10.1007/978-0-387-09595-0_12

[6] Gatfield J, Albrecht I, Zanolari B, Steinmetz MO, Pieters J (2005) Association of the leukocyte plasma membrane with the actin cytoskeleton through coiled coil-mediated trimeric coronin 1 molecules. Mol Biol Cell 16: 2786–2798. 15800061 10.1091/mbc.E05-01-0042 PMC1142424

[7] Jayachandran R, Gatfield J, Massner J, Albrecht I, Zanolari B, et al (2008) RNA interference in J774 macrophages reveals a role for coronin 1 in mycobacterial trafficking but not in actin-dependent processes. Mol Biol Cell 19: 1241–1251. 18162581 10.1091/mbc.E07-07-0640 PMC2262969

[8] Humphries CL, Balcer HI, D'Agostino JL, Winsor B, Drubin DG, et al (2002) Direct regulation of Arp2/3 complex activity and function by the actin binding protein coronin. J Cell Biol 159: 993–1004. 12499356 10.1083/jcb.200206113 PMC2173993

[9] Rybakin V, Clemen CS (2005) Coronin proteins as multifunctional regulators of the cytoskeleton and membrane trafficking. Bioessays 27: 625–632. 15892111 10.1002/bies.20235

[10] Suzuki K, Nishihata J, Arai Y, Honma N, Yamamoto K, et al (1995) Molecular cloning of a novel actin-binding protein, p57, with a WD repeat and a leucine zipper motif. FEBS Lett 364: 283–288. 7758584 10.1016/0014-5793(95)00393-n

[11] Nal B, Carroll P, Mohr E, Verthuy C, Da Silva MI, et al (2004) Coronin-1 expression in T lymphocytes: insights into protein function during T cell development and activation. Int Immunol 16: 231–240. 14734608 10.1093/intimm/dxh022

[12] Foger N, Rangell L, Danilenko DM, Chan AC (2006) Requirement for coronin 1 in T lymphocyte trafficking and cellular homeostasis. Science 313: 839–842. 16902139 10.1126/science.1130563

[13] Shiow LR, Roadcap DW, Paris K, Watson SR, Grigorova IL, et al (2008) The actin regulator coronin 1A is mutant in a thymic egress–deficient mouse strain and in a patient with severe combined immunodeficiency. Nat Immunol 9: 1307–1315. 18836449 10.1038/ni.1662 PMC2672406

[14] Mueller P, Massner J, Jayachandran R, Combaluzier B, Albrecht I, et al (2008) Regulation of T cell survival through coronin-1–mediated generation of inositol-1, 4, 5-trisphosphate and calcium mobilization after T cell receptor triggering. Nat Immunol 2008 9: 424–431. 10.1038/ni1570 18345003

[15] Mugnier B, Nal B, Verthuy C, Boyer C, Lam D, et al (2008) Coronin-1A links cytoskeleton dynamics to TCR alpha beta-induced cell signaling. PLoS One 3: e3467. 18941544 10.1371/journal.pone.0003467 PMC2568942

[16] Grogan A, Reeves E, Keep N, Wientjes F, Totty NF, et al (1997) Cytosolic phox proteins interact with and regulate the assembly of coronin in neutrophils. J Cell Sci 110: 3071–3081. 9365277 10.1242/jcs.110.24.3071

[17] Allen LAH, DeLeo FR, Gallois A, Toyoshima S, Suzuki K, et al (1999) Transient association of the nicotinamide adenine dinucleotide phosphate oxidase subunits p47phox and p67phox with phagosomes in neutrophils from patients with X-linked chronic granulomatous disease. Blood 93: 3521–3530. 10233905

[18] Tanigawa K, Suzuki K, Kimura H, Takeshita F, Wu H, et al (2009) Tryptophan aspartate-containing coat protein (CORO1A) suppresses Toll-like receptor signalling in Mycobacterium leprae infection. Clin Exp Immunol 156: 495–501. 19438603 10.1111/j.1365-2249.2009.03930.x PMC2691979

[19] Seto S, Tsujimura K, Koide Y (2012) Coronin-1a inhibits autophagosome formation around Mycobacterium tuberculosis-containing phagosomes and assists mycobacterial survival in macrophages. Cell Microbiol 14: 710–727. 22256790 10.1111/j.1462-5822.2012.01754.x

[20] Ferrari G, Langen H, Naito M, Pieters J (1999) A coat protein on phagosomes involved in the intracellular survival of mycobacteria. Cell 97: 435–447. 10338208 10.1016/s0092-8674(00)80754-0

[21] Jayachandran R, Sundaramurthy V, Combaluzier B, Mueller P, Korf H, et al (2007) Survival of mycobacteria in macrophages is mediated by coronin 1-dependent activation of calcineurin. Cell 130: 37–50. 17632055 10.1016/j.cell.2007.04.043

[22] Kielstein P, Rapp-Gabrielson VJ (1992) Designation of 15 serovars of Haemophilus parasuis on the basis of immunodiffusion using heat-stable antigen extracts. J Clin Microbiol 30: 862–865. 1572971 10.1128/jcm.30.4.862-865.1992 PMC265175

[23] Amano H, Shibata M, Kajio N, Morozumi T (1994) Pathologic observations of pigs intranasally inoculated with serovar 1, 4 and 5 of Haemophilus parasuis using immunoperoxidase method. J Vet Med Sci 56: 639. 7999883 10.1292/jvms.56.639

[24] Oliveira S, Pijoan C (2004) Haemophilus parasuis: new trends on diagnosis, epidemiology and control. Vet Microbiol 99: 1–12. 15019107 10.1016/j.vetmic.2003.12.001

[25] Chen Y, Jin H, Chen P, Li Z, Meng X, et al (2012) Haemophilus parasuis infection activates the NF-kappaB pathway in PK-15 cells through IkappaB degradation. Vet Microbiol 160: 259–263. 22704560 10.1016/j.vetmic.2012.05.021

[26] Constantoulakis P, Filiou E, Rovina N, Chras G, Hamhougia A, et al (2010) In vivo expression of innate immunity markers in patients with mycobacterium tuberculosis infection. BMC Infect Dis 10: 243. 20718957 10.1186/1471-2334-10-243 PMC2931512

[27] Akira S, Uematsu S, Takeuchi O (2006) Pathogen recognition and innate immunity. Cell 124: 783–801. 16497588 10.1016/j.cell.2006.02.015

[28] Wang Y, Liu C, Fang Y, Liu X, Li W, et al (2012) Transcription analysis on response of porcine alveolar macrophages to Haemophilus parasuis. BMC Genomics 13: 68. 22330747 10.1186/1471-2164-13-68 PMC3296652

[29] Liu XD, Chen HB, Tong Q, Li XY, Zhu MJ, et al (2011) Molecular characterization of caveolin-1 in pigs infected with Haemophilus parasuis. J Immunol 186: 3031–3046. 21282513 10.4049/jimmunol.0902687

[30] Frandoloso R, Martinez-Martinez S, Gutierrez-Martin CB, Rodriguez-Ferri EF (2012) Haemophilus parasuis serovar 5 Nagasaki strain adheres and invades PK-15 cells. Vet Microbiol 154: 347–352. 21839589 10.1016/j.vetmic.2011.07.022

[31] Engler A, Roy S, Sen CK, Padgett DA, Sheridan JF (2005) Restraint stress alters lung gene expression in an experimental influenza A viral infection. J Neuroimmunol 162: 103–111. 15833365 10.1016/j.jneuroim.2005.01.017

[32] Schiotz BL, Jorgensen SM, Rexroad C, Gjoen T, Krasnov A (2008) Transcriptomic analysis of responses to infectious salmon anemia virus infection in macrophage-like cells. Virus Res 136: 65–74. 18534703 10.1016/j.virusres.2008.04.019

[33] Purcell MK, Marjara IS, Batts W, Kurath G, Hansen JD (2011) Transcriptome analysis of rainbow trout infected with high and low virulence strains of infectious hematopoietic necrosis virus. Fish Shellfish Immunol 30: 84–93. 20883797 10.1016/j.fsi.2010.09.007

[34] Chen H, Lunney JK, Cheng L, Li X, Cao J, et al (2011) Porcine S100A8 and S100A9: molecular characterizations and crucial functions in response to Haemophilus parasuis infection. Dev Comp Immunol 35: 490–500. 21185856 10.1016/j.dci.2010.11.017

[35] Medzhitov R (2007) Recognition of microorganisms and activation of the immune response. Nature 449: 819–826. 17943118 10.1038/nature06246

[36] Segales J, Domingo M, Solano GI, Pijoan C (1997) Immunohistochemical detection of Haemophilus parasuis serovar 5 in formalin-fixed, paraffin-embedded tissues of experimentally infected swine. J Vet Diagn Invest 9: 237–243. 9249161 10.1177/104063879700900303

[37] Bouchet B, Vanier G, Jacques M, Gottschalk M (2008) Interactions of Haemophilus parasuis and its LOS with porcine brain microvascular endothelial cells. Vet Res 39: 42. 18387279 10.1051/vetres:2008019

[38] Bouchet B, Vanier G, Jacques M, Auger E, Gottschalk M (2009) Studies on the interactions of Haemophilus parasuis with porcine epithelial tracheal cells: limited role of LOS in apoptosis and pro-inflammatory cytokine release. Microb Pathog 46: 108–113. 19013513 10.1016/j.micpath.2008.10.008

[39] Schmitz F, Mages J, Heit A, Lang R, Wagner H (2004) Transcriptional activation induced in macrophages by Toll-like receptor (TLR) ligands: from expression profiling to a model of TLR signaling. Eur J Immunol 34: 2863–2873. 15368303 10.1002/eji.200425228

[40] Wan F, Lenardo MJ (2010) The nuclear signaling of NF-kappaB: current knowledge, new insights, and future perspectives. Cell Res 20: 24–33. 19997086 10.1038/cr.2009.137 PMC3052775

[41] Wilkinson JM, Sargent CA, Galina-Pantoja L, Tucker AW (2010) Gene expression profiling in the lungs of pigs with different susceptibilities to Glasser's disease. BMC Genomics 11: 455. 20670446 10.1186/1471-2164-11-455 PMC3017779

[42] Dinarello CA (2010) Anti-inflammatory Agents: Present and Future. Cell 140: 935–950. 20303881 10.1016/j.cell.2010.02.043 PMC3752337

